# A Course of Clinical Lectures, Delivered at the Hôtel-Dieu, Paris, for the Session 1842-’43

**Published:** 1843-02-04

**Authors:** A. F. Chomel

**Affiliations:** Paris


### THE MEDICAL EXAMINER,

### And Retrospect of the Medical Sciences.

Vol. VI.] PHILADELPHIA, SATURDAY, FEBRUARY 4, 1843. [No. 2.

### A COURSE OF CLINICAL LECTURES,

*Delivered at the Hôtel Dieu, Paris, for the Session 1842–'43.*

BY A. F. CHOMEL, M. D.

### LECTURE I.

[Translated for the Medical Examiner.]

CASE I. *Chronic Meningitis, with slight Hemiplegia—Symptoms of Compression—Death—Considerable Serous Effusion in the Ventricles—Anatomical Characters of Meningitis.*—At No. 11, of the Salle St. Bernard, there is a woman, aged twenty-five years, who entered the hospital on the 25th of September, for an engorgement of the breasts during lactation. Her mammæ were voluminous and hard; her pulse was frequent, and there was considerable heat of skin. On close examination you discovered something unnatural in her expression. Her manner was stupid, and before long she began to complain of intense headaches. Poultices and a suitable diet dissipated the engorgement; the fever diminished, but the cephalalgia, of which she had complained from the beginning, still persisted, and Dr. Barth, who had charge of my service at the time, directed his attention to the brain. His treatment at first was simply expectant, as he had yet been unable to form any definite opinion of the exact nature of the disease. I shall now read to you the history and progress of the case since that time.

Oct. 18th. Cephalalgia; pupils dilated; increased stupour; answers slowly and without precision; slight delirium during the night. Up to this period the progress of the symptoms has been very slow.

Oct. 24th. Vomiting last night; skin dry and hot; slight delirium with chills; severe pain in the head; two stools; respiration accelerated; on sitting up to be ausculted complained bitterly and shrieked. The state of the lungs offers nothing of note. Leeches were applied to the mastoid processes, and an alum gargle ordered on account of a slight stomatitis, and sore throat, just appearing.

Oct. 26th. Salivation has occurred. Copious mucous secretions in cerebral disease may be regarded as rather a favorable occurrence, as they act as revulsives. Slight spitting of blood; slight alteration of expression; the chin seems drawn to one side; the tip of the tongue inclines a little to the left side; same stupid, dull air.

Oct. 27th. Salivation continues. Dr. Barth would not interfere for the reasons just mentioned. The patient is still heavy and stupid; the digestive functions are good; no involuntary dejections; she is more quiet than the preceding days.

Oct. 30th. Continued vomiting; sensibility of the limbs more and more dull; motion continues, but with considerable feebleness. Continual stupor, from which she can only be roused by severe pinching.

During the first days of November, the period at which I resumed the charge of the wards, there was double strabismus, and the right pupil was very much dilated. Emaciation proceeded very rapidly, and the patient fell into a collapsed and comatose condition, from which she was only aroused to give utterance to some vague and inarticulate expressions; habitually she remained in a complete state of immobility; the head thrown to one side, the eye haggard, with a completely besotted expression. The pulse became more and more small; the skin, particularly of the extremities, cold and of a purple hue; sensibility more and more obtuse, so that you might pinch her without causing any sensation. The vomiting ceased, but the stools became involuntary and continued. All the symptoms progressively increased, and the patient sank on the 24th November, about three o'clock in the afternoon.

Now the train of symptoms, continued Dr. Chomel, which this patient presented when I resumed the service, led me to infer a chronic cerebral affection, but I at first hesitated between a softening of the brain, a tumour, or a meningitis; but I inclined to the belief that it was the latter. The slight hemiplegia of the left side might, it is true, induce us to suspect the existence of a tumour in the right lobe of the brain, but it was at the same time very slight, and was rather a rigidity of the limbs, or muscular contraction, than genuine paralysis. It is besides very common to see these symptoms follow true meningitis, and they are not difficult to explain. I have had occasion to observe a large number of cases of acute meningitis, which offered us during life this marked difference in the degree of contractility of the two sides, and which revealed, on examination after death, the ordinary lesions of meningitis, with more or less serous effusion in the ventricles, without however any great difference in the pathological alterations of the two sides of the brain. It is very probable that the paralysis observed during life was produced by unequal compression, the serous effusion being more abundant on one side than the other. The paralysis, in fact, went on diminishing in proportion to the approach of death, or, to express it better, as the serous effusion became diffused over a larger portion of the cerebral substances, and the compression became, in consequence, more uniform; a species of general collapse succeeding to the hemiplegic contraction. This was the case with our patient, for the semi-paralysis which she experienced at the beginning of the disease successively diminished with its progress. The autopsy showed us the cerebral convolutions very much flattened, as if they had been long subjected to considerable compression. This aspect of the exterior of the brain enabled us to predict the existence of serous effusion in the ventricles. On opening the brain we found, in fact, four or five teaspoonfuls of serum in the two ventricles. The serum was yellow, transparent, and unmixed with any albuminous flocculi. The pia-mater was normal, and was readily detached; the arachnoid was sticky and adhered to the finger; the cerebral matter was firm, even more so than natural. There was no intracranial tumour. Thus in every point did the autopsy confirm our diagnosis.

With this case I will connect another which has some points of resemblance.

CASE II.—*Partial Hemiplegia of left side.—Paralysis of the right superior lid, and of the motor muscles of the eye.—The consequence of repeated epileptic at-*

*tacks.*—At No. 12, in the same ward, is a woman affected with incomplete paralysis of the left arm, with paralysis of the right superior eyelid, and of the internal, superior, and inferior recti muscles of the left eye, for the globe is in a constant state of forced abduction, and can execute no movement upwards, downwards, or inwards; it remains in a state of nearly immoveable diverging strabismus. Such was her condition on entering the hospital.

Let us now examine what can produce these phenomena. What is the disease in fact under which she is laboring? This woman says, that, for nearly three years she has been subject to epileptic fits. It is possible that she even had them before she was aware of the fact; we will, however, bear in mind, what she has told us concerning them. After the first attack she felt in all the left side a species of numbness, which by degrees disappeared, and sensation became natural. Since her infancy she had been subject to headache, which affected more particularly the right side. Other epileptic attacks followed, principally at the menstrual periods; they were preceded by no prodromes, seizing her suddenly, wherever she might be, without her being able to prevent or retard them. This latter circumstance is by no means unimportant, for it is one of the means of distinguishing epilepsy from hysteria. Hysterical women are, as every body knows, subject to epileptiform convulsions, but these convulsions are very different from those of our patient. Hysterical fits are never so sudden as those of epilepsy; they are to a certain extent subject to the will; while those of epilepsy are entirely beyond its influence. Hysterical women can, in a degree, control their attacks, and hence they rarely occur in the street, or in public places; indeed, they rarely have them, except when surrounded by those whose interest and sympathy they know will be excited. Hence, whenever you see a woman fall in the street in a fit, you may be assured that it is epilepsy and not an hysterical convulsion.

Let us return to our patient. Two or three months ago she was suddenly taken with one of these attacks in the street. During its access the extremities of the left side of the body became instantaneously paralyzed, and in this condition she was brought to the hospital. At the time of admission there was some diminution in the paralysis of the inferior member. She was unable to give any account of herself, as she remained immovable in bed, with nearly an entire loss of intelligence, and answering only vaguely and incoherently to questions, so that the degree of the paralysis of the limbs might be equivocal. The cessation of the paralysis in the limb, if real, was not of any great value in the diagnosis; for in cerebral affections of the same nature, the superior members sometimes are alone paralysed, whilst at other times the superior and inferior are equally affected. To complete the group of symptoms offered by this patient, let it be remembered that the mouth was inclined to the left side, that she screamed violently, and fell into a complete state of delirium, talking incessantly during the night, whilst during the day she was quiet enough.

What is the cerebral affection which can produce and maintain this train of phenomena? It can be readily conceived that a hemorrhage, for example, occurring on the side opposite to the existing lesions could, by compressing that side of the brain, produce the greater part of these symptoms. But a hemorrhage, as well as ramollissment, could only cause these symptoms once. When these convulsive movements, these epileptiform attacks occur frequently at short intervals, you must seek some other cause of disease—a tumour for example. The most common organic cause of epilepsy are tumours of different kinds, situated in the cerebral mass. Hence it is in that direction that we must look for the exciting cause. If at the commencement of the disease there had been actual true paralysis, not followed by subsequent attacks, we could have admitted a cerebral hemorrhage; but we had an attack followed by a slight numbness and torpor in the members of the left side, then a return to a normal condition, and finally a last attack, producing the more marked and permament symptoms that we now observe. This is precisely the ordinary progress of symptomatic epilepsy; that is to say, of that variety which is caused by a material organic alteration—a tumour, for example. Sometimes these cerebral tumours remain stationary, and then the consequent paralysis disappears gradually, to again reappear when the exciting cause makes fresh progress. It is thus that we are enabled to explain the intervals of quiet which patients suffering from this disease present, and of which the present case in particular offers a striking example. The time however arrives when the development of the disease is hastened, and a fatal termination soon follows.

The diagnosis of cerebral disease is always more doubtful than that of any other class of diseases—pulmonary affections for example; for the difficulty of diagnosis, in this case, is in proportion to the difficulty of exploring the diseased organ. We can only guide ourselves by the apparent symptoms, and the probable causes of the disease. Hence we are very far from being sure of the correctness of our diagnosis in the present case; but there is great probability that it is produced by a material alteration of the brain; by a tumour located, from all appearances, in the right hemisphere.

The prognosis, as you may imagine, is very grave; the termination of the disease is not doubtful; it is irrevocably mortal; the exact period of its termination is only uncertain. The patient may live, or rather vegetate for some time; but sooner or latter she must succumb. It is possible she will yet suffer from future attacks, in one of which she will die, or that softening will occur around the tumour, which disorganizing more or less the brain, will produce death.

As to treatment, there is none that is efficacious. The resources of art are reduced to palliatives and other means to retard the progress of the disease; such as setons behind the ear, mercurial frictions at the back of the neck, blisters and sinapisms on the extremities, purgatives, opiates, &c. &c. These are the only means that the materia medica places at our disposal to combat a similar disease.

*Paris, Dec.* 1842.

